# Proposal and proof-of-principle demonstration of non-destructive detection of photonic qubits using a Tm:LiNbO_3_ waveguide

**DOI:** 10.1038/ncomms13454

**Published:** 2016-11-17

**Authors:** N. Sinclair, K. Heshami, C. Deshmukh, D. Oblak, C. Simon, W. Tittel

**Affiliations:** 1 Department of Physics and Astronomy, Institute for Quantum Science and Technology, University of Calgary, Calgary, Alberta, Canada T2N 1N4; 2 National Research Council of Canada, 100 Sussex Drive, Ottawa, Ontario, Canada K1A 0R6

## Abstract

Non-destructive detection of photonic qubits is an enabling technology for quantum information processing and quantum communication. For practical applications, such as quantum repeaters and networks, it is desirable to implement such detection in a way that allows some form of multiplexing as well as easy integration with other components such as solid-state quantum memories. Here, we propose an approach to non-destructive photonic qubit detection that promises to have all the mentioned features. Mediated by an impurity-doped crystal, a signal photon in an arbitrary time-bin qubit state modulates the phase of an intense probe pulse that is stored during the interaction. Using a thulium-doped waveguide in LiNbO_3_, we perform a proof-of-principle experiment with macroscopic signal pulses, demonstrating the expected cross-phase modulation as well as the ability to preserve the coherence between temporal modes. Our findings open the path to a new key component of quantum photonics based on rare-earth-ion-doped crystals.

## Abstract (web summary)

Rare-earth doped crystals are a promising platform for developing quantum devices. Here, Sinclair *et al*. propose and demonstrate a concept for non-destructive detection of photonic qubits using solid-state waveguides, which could help reduce signal losses in quantum information processing.

## Graphical abstract

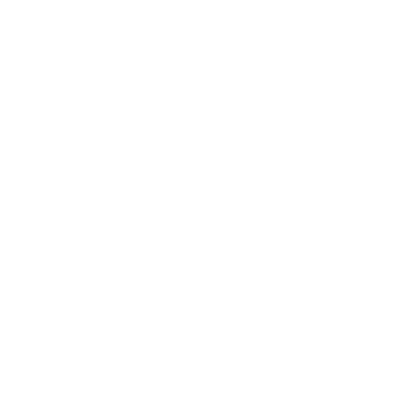

The possibility to detect the presence of a visible or telecommunication-wavelength photon in a non-destructive^1^ and state-preserving manner is highly desirable for photonic quantum information processing^2^ and quantum networks^3^,^4^,^5^ as it makes it possible to use precious resources (say entangled photon pairs for quantum teleportation) only when required (for example, if the signal photons whose state is to be teleported are actually there). This is all the more essential in cases where significant signal loss occurs, for example, in quantum repeaters^5^,^6^. As such, several approaches to non-destructive photon and, exceptionally, non-destructive qubit detection are currently being pursued. For instance, non-destructive detection of photons^7^ and heralded storage of photonic qubits^8^ (which, when combined with readout, is equivalent to non-destructive qubit detection) have been achieved using single, laser-trapped atoms in high-finesse cavities. Non-destructive detection of optical photons is also being pursued with atomic ensembles, and large single-photon phase shifts mediated by laser-cooled atomic vapour have recently been reported using both Rydberg blockade^9^ and the a.c. Stark shift in a high-finesse cavity^10^. The latter system has furthermore enabled partial non-destructive detection of traveling photons^11^. These investigations are part of the general drive of using atomic ensembles to mediate strong photon–photon interactions^12^,^13^, for example, via strong long-range dipole–dipole interaction between Rydberg atoms^14^ or via the a.c. Stark shift^15^,^16^. Experimental realizations of photon–photon interaction using Rydberg states^17^,^18^,^19^,^20^, and the a.c. Stark shift^21^,^22^,^23^,^24^,^25^,^26^ have furthermore enabled applications such as all-optical switching^27^,^28^,^29^,^30^. Finally, we note that non-destructive detection has been achieved for microwave photons inside cavities using superconducting circuits^31^ and Rydberg atoms^32^,^33^,^34^. Currently, the most advanced experiments targeting the detection of optical photons rely on single atoms or cold atomic gases. However, from the point of view of practical applications, it is of interest to investigate implementations in the solid state as well. Ideally, such approaches should preserve the qubit state encoded into the photon, allow for multiplexing and be compatible with existing solid-state quantum information processing and communication components.

Here, we propose a scheme for non-destructive detection of photonic time-bin qubits that has all of these characteristics. After a short theoretical description of our scheme, we detail a proof-of-principle experiment using intense coherent pulses and an impurity-doped crystal that confirms the predictions of our theory. Finally, we discuss how our proposal can be implemented at the single-photon level.

## Results

### Proposal for quantum non-destructive measurement

**Figure 1:**
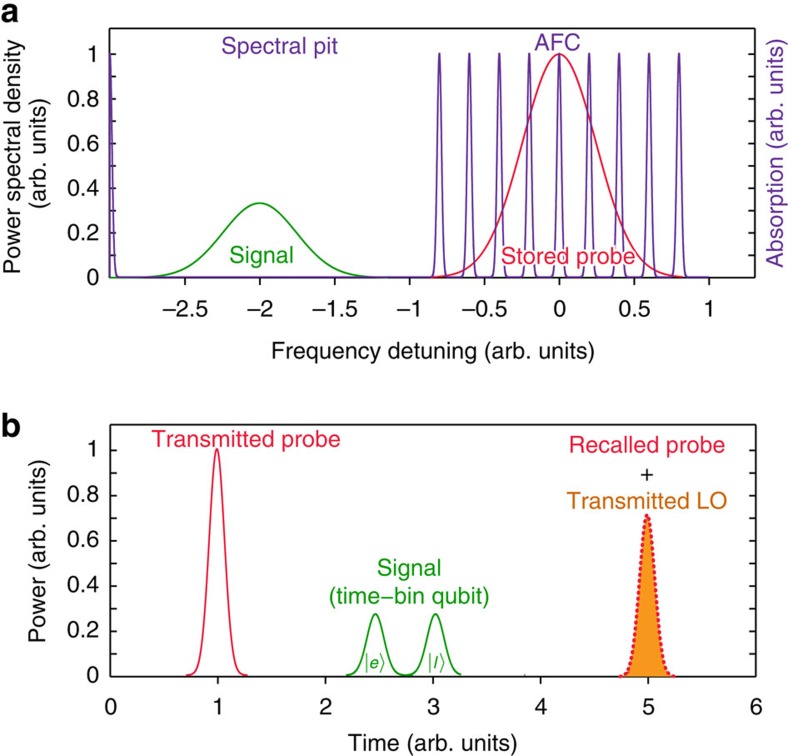
Protocol for non-destructive detection of photonic time-bin qubits. A macroscopic probe pulse is stored in an AFC. The signal—a photonic time-bin qubit—propagates through a detuned transparency window and frequency-shifts the atoms constituting the AFC due to the a.c. Stark effect. This results in a phase shift of the re-emitted probe. A spectral representation of our protocol is shown in **a**, while a temporal representation is shown in **b**. The time-bin qubit states |*e*〉 and |*l*〉 refer to early and late temporal modes, respectively.

The basic principle of our scheme, illustrated in Fig. 1, is based on cross-phase modulation between a weak signal and a strong probe pulse mediated by a rare-earth-ion-doped crystal—a technology platform whose suitability for quantum photonics has already been demonstrated^35^,^36^,^37^,^38^,^39^,^40^,^41^,^42^. For single-photon sensitivity, the phase shift has to be greater than the quantum phase uncertainty of the probe, which is of order 1/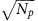, where *N*_*p*_ is the number of photons in the probe. The probe is stored in an impurity-doped crystal using an approach based on atomic frequency combs^43^, and the phase shift is due to the a.c. Stark shift of the relevant atomic transition caused by the propagating signal. For large detuning between signal and probe, it is given by


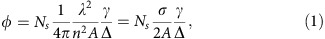


where, *N*_*s*_ is the number of photons in the signal, *λ* is the vacuum wavelength of the atomic transition, *n* is the refractive index of the crystal, *A* is the transverse mode area of the interaction, *γ* is the spontaneous decay rate from the excited state, Δ/(2*π*) is the detuning in Hz and *σ*=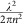 is the resonance absorption cross-section of a radiatively broadened transition. See Supplementary Note 1 and Supplementary Fig. 1 for a detailed derivation. Equation (1) shows that the phase shift benefits from lateral confinement (small *A*) and small detuning, and that it increases linearly with the number of signal photons.

We emphasize that the phase shift does not depend on the exact timing of the signal, as long as it propagates through the medium, while the probe is being stored. In particular, this allows one to detect the presence of a photon without affecting its qubit state, provided that the qubit is encoded in temporal modes^44^, a very convenient and widely-used choice in quantum communication. (Note that photonic qubits can easily be converted between different types of encoding^45^).

### Proof-of-principle demonstration using strong coherent pulses

**Figure 2:**
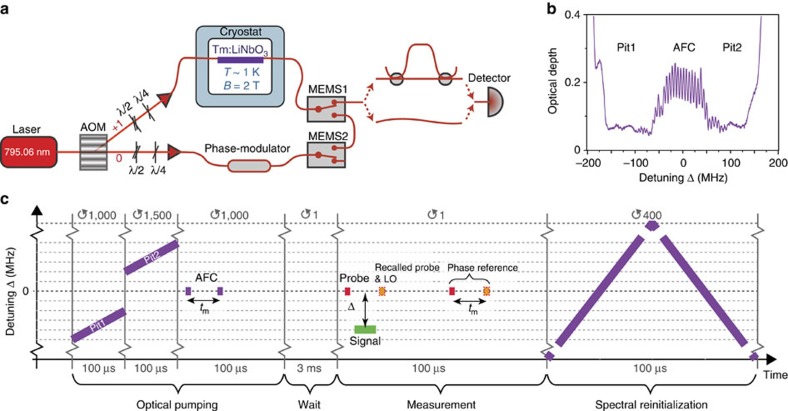
Outline of the experiment. (**a**) Setup. Light from a frequency-locked 795.06 nm CW-laser is intensity and frequency modulated using an acousto-optic modulator (AOM). The diffracted first-order beam is coupled via a fibre into the Tm:LiNbO_3_ waveguide, and waveplates enable adjusting its polarization to maximize the interaction with Tm ions within a single spatial mode of ∼12.5 μm diameter (characterized independently). (**b**) Spectral feature. A 100 MHz wide AFC with a tooth separation Δ_*m*_/(2*π*)=5.5 MHz (corresponding to a storage time of *t*_*m*_=180 ns) and a 100 MHz wide spectral pit on either side of the AFC. (**c**) Timing sequence. Optical pumping involves repetitive spectral pit burning at negative (−150 to −50 MHz) and positive (50 to 150 MHz) detunings for a total of 250 ms, and AFC generation using many pulse-pairs for 100 ms. (Depicted is one repetition, while the number following the circular arrow indicates the repetitions per task). After a 3 ms wait time to allow the excited atomic population to decay, we perform our measurement: A 10 ns long probe is stored in the AFC, followed by a detuned signal that is transmitted through a spectral pit. A LO interferes with the probe pulse recalled after 180 ns storage. Another 200 ns later, we perform a phase reference measurement using the same sequence but excluding the signal pulse. At the waveguide output, a micro electro-mechanical switch (MEMS1) blocks light during optical pumping. It opens during the measurement to allow the transmission of the recalled probe pulse to the detector—either directly or via an unbalanced interferometer, depending on the measurement performed. As the strong probe pulses modify the tailored spectral feature, we re-initialize the absorption line after every measurement using zeroth-order light from the AOM that is repetitively frequency-modulated over a 5-GHz range by a phase modulator. The light enters the Tm-doped waveguide through MEMS2 and MEMS1; it is blocked by MEMS2 outside the reinitialization step of 40 ms duration.

Our experimental setup, sketched in Fig. 2a, is composed of a Tm:LiNbO_3_ waveguide, a source for signal and probe pulses, and analyzers that allow characterizing these pulses after the waveguide-mediated interaction. We use the optical pumping sequence illustrated in Fig. 2b to spectrally tailor the inhomogeneously broadened ^3^*H*_6_→^3^*H*_4_ absorption line of Tm into a series of absorption peaks (teeth) spaced by angular frequency Δ_*m*_, an atomic frequency comb (AFC), surrounded by transparent pits (see Fig. 2c). The bandwidth of the AFC and each of the pits is about 100 MHz, and the storage time of the probe in the waveguide, given by *t*_*m*_=2*π*/Δ_*m*_, is 180 ns.

Following the spectral tailoring, we generate a probe pulse of ∼10 ns duration whose spectrum matches the AFC. A part of the pulse is transmitted through the waveguide and a part of it is stored in the thulium ions that form the AFC. As illustrated in Fig. 2b, we then send a signal whose temporal structure, intensity and detuning with respect to the AFC we can vary, depending on the desired measurement. After the storage time *t*_*m*_ the probe pulse is re-emitted from the waveguide. To measure its phase change due to the interaction with the signal, we interfere it with a local oscillator (LO). See the ‘Methods' section for more details about AFC generation and measurement.

**Figure 3:**
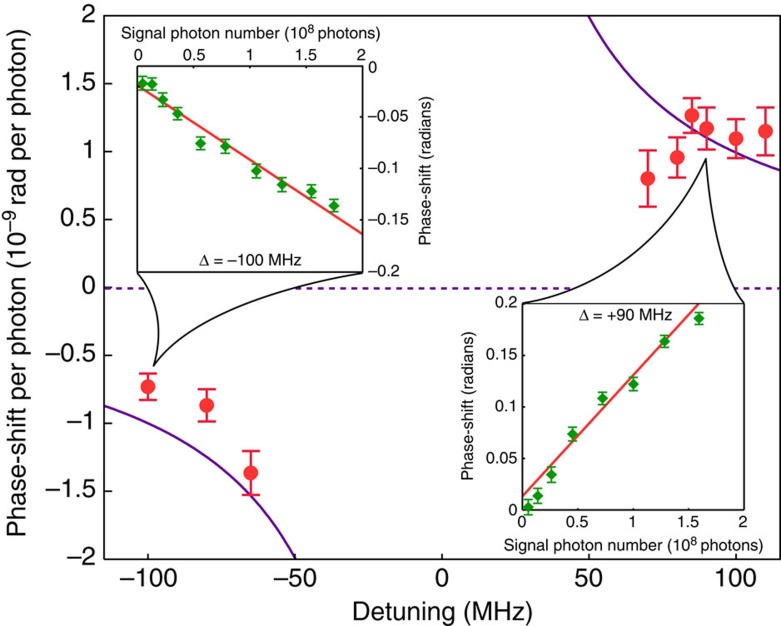
Phase shift per photon for different detuning values. Expected phase shifts (purple line) calculated using equation 1 with independently measured quantities (no fit), and experimentally obtained values (red circles) derived from linear fits to the phase shift versus mean photon number as illustrated in the insets for two detuning values (red lines). Uncertainty bars on the red data points are based on the s.d. of the slope from the linear fits. Each data point in the insets (green diamonds) corresponds to an average over 200 repetitions and the uncertainty bars indicate the s.d. of the average. Discrepancies between measured and predicted values are most likely caused by (too) short acousto-optic modulator (AOM) drive signals featuring a spectral width that approaches or exceeds the AOM's bandwidth limit, which results in imperfections of both the signal pulse spectra and the tailored atomic absorption spectrum.

First, to verify the probe-phase-shift dependence given in equation 1, we use a signal pulse in a single temporal mode of 130 ns duration. We vary the number of photons per pulse for nine different detunings, and record the phase shift of a probe pulse featuring a mean photon number of 1.7 × 10^9^, averaged over 200 repetitions, for each type of signal pulse. See Supplementary Fig. 2 for an example of the detected output with and without the signal present. As expected, we find a linear increase as a function of the number of signal photons, and that the slopes for red and blue detuning have opposite signs, as shown for two detunings in the insets of Fig. 3. From the fitted slopes we find the phase-shifts per photon for different detunings, which are shown in Fig. 3 together with the expected values. We see that the measured data closely follow the theoretical predictions derived from equation 1 using *λ*=795 nm, *n*=2.3, *A*=*π* × (6.25 μm)^2^, *γ*=8.1 kHz. In particular, at +100 MHz detuning, we measure a phase shift of (1.10±0.14) × 10^−9^ rad per photon, which is in good agreement with the expected value of 1.0 × 10^−9^ rad per photon. We note that there is some uncertainty on the parameters—such as the waveguide cross-sectional area and fibre-to-waveguide coupling loss—that go into this estimate.

**Figure 4:**
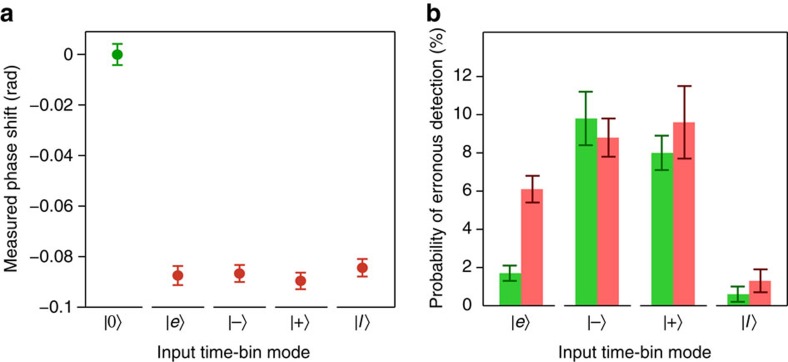
State preservation for different signal pulses. (**a**) Probe phase shifts due to 6.9 × 10^7^, or no, signal photons, distributed between early and late temporal modes. The labels on the *x*-axis refer to either no signal photons (|0〉) or the corresponding time-bin qubit states, where |*e*〉 and |*l*〉 refer to qubits prepared in early and late temporal modes, respectively, and |+〉 and |−〉 represent the superposition states (|*e*〉+|*l*〉)/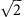 and (|*e*〉−|*l*〉)/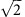, respectively. Each data point shows the average over 1,000 measurements, and uncertainty bars denote the s.d. of the average. (**b**) The error rates—the ratio of the energy detected in the wrong output mode to the total energy detected in both the correct and wrong modes—of the different signal states before (green bars) and after (red bars) the measurement. Error bars are calculated from shot-to-shot pulse-heights variations. There is no significant change, except for |*e*〉. (Increased errors are likely due to free induction decay after the signal pulse excites remaining thulium atoms inside the pit, and would disappear with better hole burning. As the decay happens after absorption, only |*e*〉 is affected. Errors for the superposition states are caused by imperfections in the interferometer).

Next, we demonstrate that the probe phase shift does not depend on how the signal energy is distributed between two temporal modes, and that the signal is not affected by the measurement. Put into the context of an interaction with a single photon in a time-bin qubit state, this implies that the measurement does not project the qubit onto a specific set of basis states and thus alter it. Towards this end, we select early and late signal modes, each of 10 ns duration, separated by 18.3 ns, and featuring a detuning of +100 MHz. Keeping the total energy constant, we generate signals in which the energy is concentrated in either the early or the late mode, or in an equal superposition with either 0 or *π* phase-difference (‘+' and ‘−' superpositions, respectively). The resulting probe phase shifts, averaged for each pulse sequence over 1,000 repetitions, are plotted in Fig. 4, which also includes the phase shift measured without a signal pulse. We find that, within experimental uncertainty, the phase shifts are the same irrespective of the signal state, and that they clearly differ from the phase shift measured without any signal. Furthermore, to verify that our measurement preserves the signal state, we assess erroneous detections of signals prepared in various states without and with the cross-phase interaction measurement (see the ‘Methods' section for details). As shown in the inset of Fig. 4, we find close to no change due to the cross-phase interaction, which is consistent with the fact that our scheme can measure the presence of a time-bin qubit without revealing, or modifying, its state.

## Discussion

While our proof-of-principle demonstration confirms the key features of the proposed scheme, a lot remains to be done before qubits encoded into individual and spectrally multiplexed photons can be detected non-destructively and without averaging. We expect that a reduction of the interaction cross section, for example, using a small-diameter ridge waveguide or structures fabricated by focused-ion-beam milling^46^,^47^, can improve the phase sensitivity by more than a factor of 100 (see equation 1). Furthermore, the ratio between the radiative lifetime *γ* and the detuning Δ has to be increased beyond its current value of 8.1 kHz/(2*π* × 65 MHz)∼2 × 10^−5^—as we discuss below, this ratio can in principle approach one per cent. With these improvements, the phase shift per photon could thus be as large as 0.1 mrad, which would allow single-shot detection of individual photons^48^.

Reducing the detuning to maximize *γ*/Δ comes with the unwanted effects of increasing off-resonant absorption of the signal in the AFC, increasing the noise due to decay from excited atoms, and decreasing the bandwidth of the signal. However, as we discuss in more detail in Supplementary Note 1, these problems can be overcome in a configuration in which the population in the excited state (populated through the absorption of the strong probe in the AFC) is temporarily transferred to an auxiliary level, and in which the signal passes many times through the spectral pit during the storage of the probe using, for example, a cavity^40^. This makes it possible to increase the detuning and thus reduce the absorption of the signal without decreasing the number of atoms in the AFC nor the total phase shift experienced by the probe. For instance, we anticipate the non-destructive measurement to be feasible using an AFC with teeth of optical depth 30 (ref. 37) and signal photons of half a MHz bandwidth that interact ∼900 times with the stored probe, which corresponds to the use of a moderate-finesse cavity (see Supplementary Note 1 for details).

We emphasize that the cross-phase interaction in rare-earth-ion-doped crystals is straightforward to generalize to multiple spectral channels, as demonstrated in the context of AFC-based optical quantum memory^41^, which can extend over a total bandwidth of hundreds of GHz^49^. We also note that the present approach should allow the development of a standard (destructive) photon-number-resolving detector, for which the limitations imposed by signal loss and noise are less severe.

We believe that an improved version of our proof-of-principle demonstration will soon allow first destructive, and then non-destructive, single-shot detection of photons. This will open the path to more efficient use of precious probabilistic resources, such as entangled photons, in advanced applications of quantum communication. Furthermore, it will allow the heralded generation of photon-number states, including entangled states, that do not contain often detrimental admixtures of undesired photons as, for example, in widely used spontaneous parametric down-conversion^50^. Finally, we note that for optimal implementation of our scheme, the bandwidth of the signal pulse depends linearly on the linewidth of the relevant atomic transition, which, in our case, is around 3 kHz (ref. 51). Hence, to allow the non-destructive detection of photons featuring more than around 1 MHz bandwidth, it may be interesting to investigate impurity-doped crystals and transitions that feature larger (but still radiatively limited) homogeneous linewidths. As an example, the 4*f*→5*d* transition in Ce^3+^:Y_2_SiO_5_ features a 3 MHz lifetime-limited homogeneous linewidth^52^, which is more than 2 orders of magnitude larger than in Tm, which we used in our current realization.

## Methods

### Spectral tailoring

We tailor the spectrum of the inhomogeneously broadened ^3^*H*_6_→^3^*H*_4_ absorption line in Tm^3+^ by means of frequency-selective optical pumping of Tm ions to another ground-state Zeeman level^49^. The difference in Zeeman splittings of the ground and excited states in the applied 2 T field is ∼2.5 GHz—it sets the upper limit for the total width of our spectral feature (the AFC and the two pits). However, the bandwidth of the AOM used for laser frequency modulation practically limits the total width to 300 MHz.

First, as illustrated in Fig. 2b, we generate two spectral pits by sweeping the frequency of the laser light repeatedly over two 100 MHz wide regions separated by a spectral interval of 100 MHz. The optical depth of the remaining background is around 0.07. It is irregular due to varying efficiency of the AOM with detuning. Next, we generate an AFC in-between the two pits by driving the AOM using pairs of pulses that are 10 ns wide and separated by 180 ns. The resulting AFC features a bandwidth of 100 MHz and a tooth separation of Δ_*m*_/(2*π*)=5.5 MHz, corresponding to a storage time of 180 ns. The tooth separation is chosen to match side-peaks at 11 MHz arising from the super-hyperfine interaction of thulium with niobium in the host crystal (N. Sinclair *et al*., manuscript in preparation), and the teeth width is limited by long-term laser-frequency drift (1 MHz) combined with power broadening during spectral tailoring. The teeth feature an optical depth of ∼0.1, and are sitting on a background with optical depth of ∼0.15, resulting in a recall efficiency for the probe of 0.2% (ref. 43).

The quality of our spectral feature—the background in the pits and the AFC, as well as the small optical depth of the AFC teeth—is currently limited by non-ideal spectral tailoring. It can be improved by using a laser with improved stability, and by optical pumping based on a burn-back method^37^. This will allow meeting the requirements for a non-destructive measurement at the single photon level as detailed in Supplementary Note 1.

### Phase measurements

Assessing the cross-phase modulation relies on an interferometric measurement of the recalled probe pulse with a transmitted LO in the same spatial, temporal and spectral mode, and featuring the same intensity. First, by varying the phase of the LO in the absence of a signal, we calibrate the interference visibility to 89.7%. Next, to ensure maximum measurement sensitivity, we set the phase difference between the LO and the recalled probe (still without a signal) to *π*/2. Taking the calibration into account, this allows us in the actual measurement to map intensities (after interfering the probe with the LO) onto phase changes of the probe. Please note that the intensity of the recalled probe does not depend on whether or not a signal is present, that is, the calibration, taken without any signal, remains valid when the latter is present.

The precision of the phase measurements is mainly limited by long-term laser frequency instability. We estimate that fluctuations between the AFC generation and the creation of the probe ∼3 ms later result in shot-to-shot noise of around 150 mrad. To reduce this noise, we concatenate each measurement of the a.c. Stark shift on the probe with a reference measurement of the probe's phase without a signal (see Fig. 2b). Subtracting the values obtained by these two phase measurements (with a weight given by the correlation of two subsequent measurements without signal) allows improving the single-shot phase sensitivity to around 100 mrad. This value is mainly limited by laser frequency fluctuations between the generation of the probe and LO pulses, and can be further reduced by improved laser locking. In addition, pulse intensity fluctuations and electronic noise of the photodetector contribute ∼50 mrad of phase uncertainty. By averaging phases over *j* measurement repetitions, the sensitivity improves by a factor of *j*^−1/2^. For instance, for *j*=200, we reach a resolution of ∼7 mrad.

### Qubit measurements

The variation of the signal due to the interaction with the probe is assessed as follows: for early and late signal states we measure the pulse heights in the wrong time bin, normalized to the sum of the pulse heights in both bins. For the superposition states, we pass the signal through an imbalanced fiber interferometer whose arm-length difference corresponds to 18.3 ns travel-time difference. Using a piezoelectric transducer in one arm of the interferometer, we set its phase to obtain maximum constructive interference in one output, and record the normalized pulse heights in the other (the wrong) output. All measurements are done twice—once before, and once after the signal is submitted to the cross-phase interaction. Differences in the results indicate the perturbation of the signal due to the measurement.

### Data availability

The authors declare that the data supporting the findings of this study are available within the article and its Supplementary Information.

## Additional information

**How to cite this article:** Sinclair, N. *et al*. Proposal and proof-of-principle demonstration of non-destructive detection of photonic qubits using a Tm:LiNbO_3_ waveguide. *Nat. Commun.* **7,** 13454 doi: 10.1038/ncomms13454 (2016).

**Publisher's note:** Springer Nature remains neutral with regard to jurisdictional claims in published maps and institutional affiliations.

## Supplementary Material

Supplementary Information Supplementary Figures 1-2, Supplementary Note 1 and Supplementary References
