# Supplementary material for: Proposal and proof-of-principle demonstration of non-destructive detection of photonic qubits using a Tm:LiNbO3 waveguide
Source: Nat Commun. 2016 Nov 17;7:13454. doi: 10.1038/ncomms13454 (PMC5118539; doi:10.1038/ncomms13454)
Supplement: Supplementary Information — Supplementary Figures 1-2, Supplementary Note 1 and Supplementary References [file ncomms13454-s1.pdf]

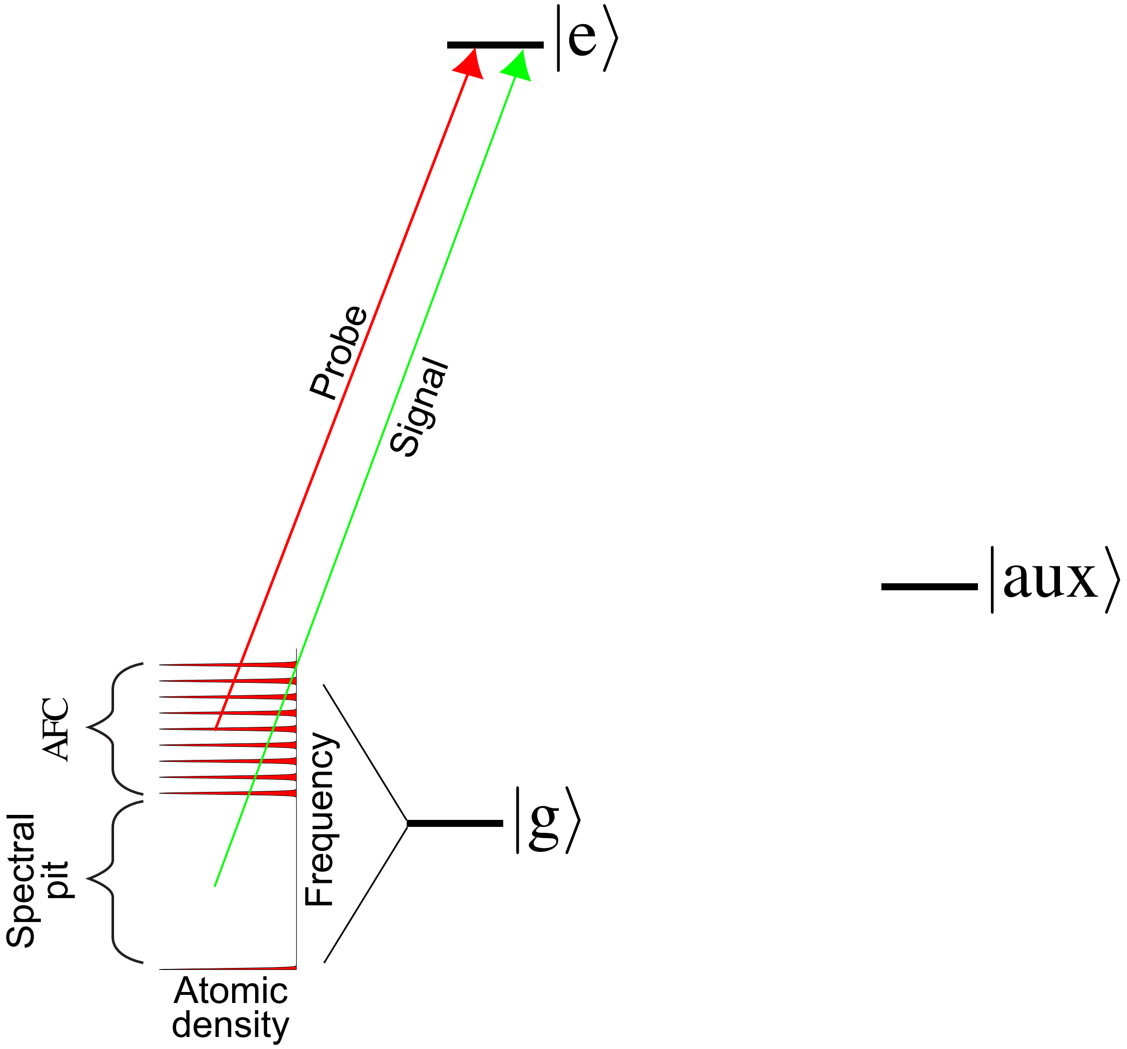

Supplementary Figure 1: Level structure for the proposed protocol. To perform cross-phase modulation of a stored probe in an inhomogeneously broadened ensemble an atomic frequency comb and a transparent spectral pit are prepared. The auxiliary state $|\mathrm{aux}\rangle$ is used for shelving in preparation of the atomic frequency comb (AFC) and spectral pit.

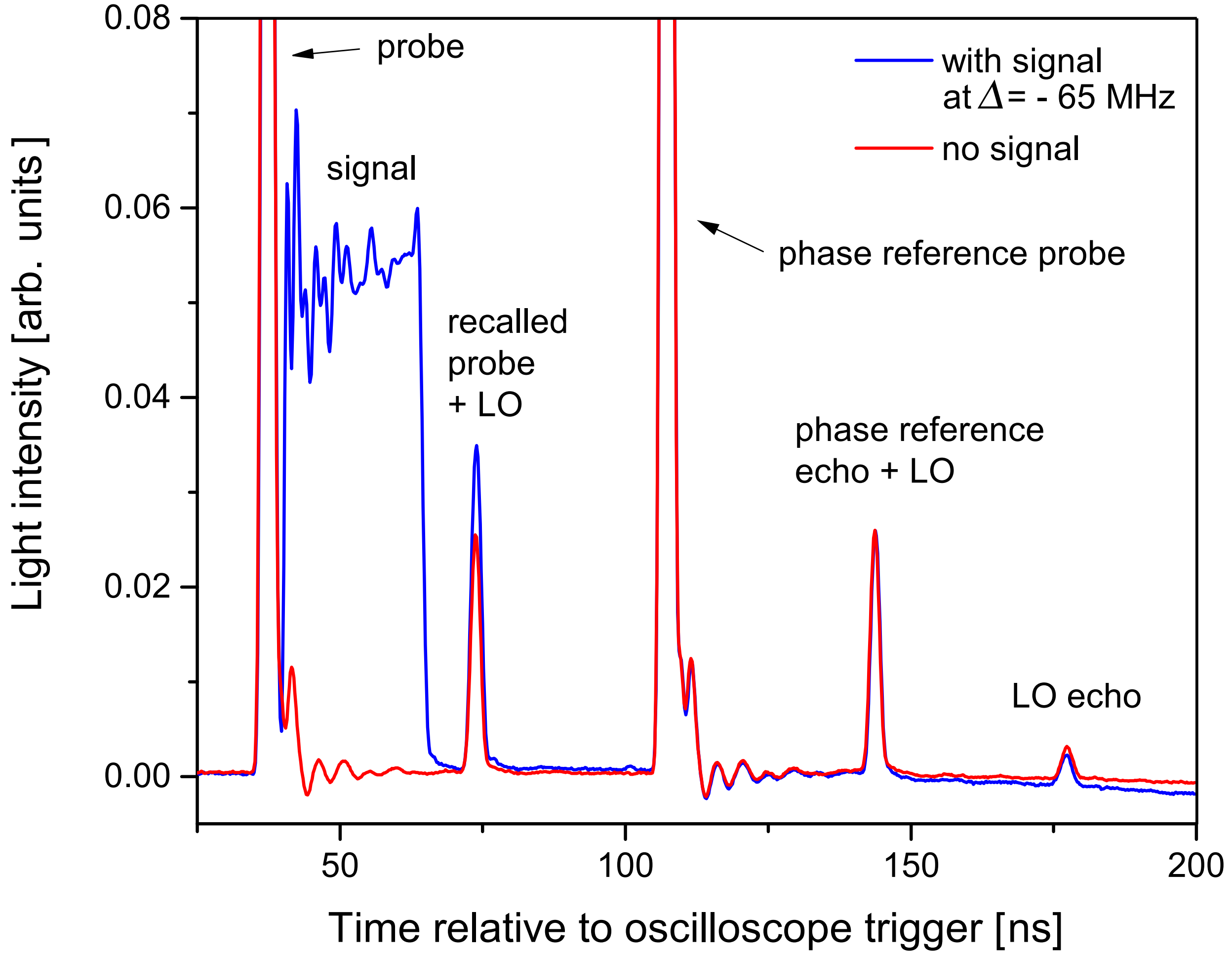

Supplementary Figure 2: Example of detected output with and without the signal pulse. In this example, the signal pulse has a duration of 130 ns, is detuned by -65 MHz, and features a mean photon number of 8×10^7. This data goes into the results shown in Fig. 2 in the main text. The displayed output traces are generated by taking an average over 200 repetitions. The output consists of a transmitted probe pulse (probe), the transmitted signal pulse (signal), the recalled probe superposed with the transmitted local oscillator (recalled probe + LO), the transmitted phase reference pulse (phase reference pulse), its respective echo that is superposed with a transmitted local oscillator (phase reference echo + LO), and the echo of the second local oscillator (LO echo). Ringing after the strong probe and reference pulses (both are truncated in the plot) is due to detector saturation. The distortion of the signal pulse is due to the response of the acousto-optic modulator being driven at a frequency that differs from its nominal value (defined by zero detuning).

Supplementary Note 1: Detailed theory of the non-destructive qubit measurement

Storage of the probe and use of additional levels. The first step in our protocol involves the storage of a classical probe field. Here, we provide a semi-classical treatment of the light-matter interaction to describe atomic frequency comb (AFC) storage and retrieval of the probe field (see also Supplementary Reference 1). The total Hamiltonian describing our system is given by

$$\hat{H} = \hat{H}_0 + \hat{H}_{\text{int}}, \tag{1}$$

where

$$\hat{H}_0 = \sum_{j=1}^{N} \hbar\omega_{\text{ge}}^j \hat{\sigma}_{\text{ee}}^j, \tag{2}$$

and

$$\hat{H}_{\text{int}} = -\hbar \sum_{j=1}^{N} \big(\mathit{\Omega}(z,t)\hat{\sigma}_{\text{eg}}^j e^{-i\omega_p(t-z_j/c)} + H.c.\big). \tag{3}$$

Here, $\hbar\omega_{\text{ge}}^j$ denotes the transition energy of atom j between ground and excited states ($|g\rangle$ and $|e\rangle$), ω_p is the control frequency of the probe field and H.c. is the Hermitian conjugate of the first term inside the summation. $\mathit{\Omega}(z,t) = \frac{\mu_{\text{eg}}\mathcal{E}_p(z,t)}{2\hbar}$ is the Rabi frequency associated with the probe field, and $\mu_{\text{eg}} = \langle e|\hat{\mathbf{d}}\cdot\boldsymbol{\epsilon}_p|g\rangle$ is the transition dipole moment, where $\mathcal{E}_p$ is the slowly varying component of the probe field $\mathbf{E}_p(z,t) = \boldsymbol{\epsilon}_p\mathcal{E}_p(t-z/c)\cos(\omega_p(t-z/c))$ with polarization unit vector of $\boldsymbol{\epsilon}_p$. The atomic coherence and population for atom j are defined by $\hat{\sigma}_{\nu\nu'}^j = |\nu\rangle^j\langle\nu'|$, where $\nu,\nu' = \{\text{g},\text{e}\}$.

We define collective atomic coherence and population operators for all atoms in a slice of the medium at (longitudinal) position z and relative resonance frequency δ as follows,

$$\hat{\sigma}_{\text{gg}}(z,t;\delta) = \frac{1}{N_z(\delta)} \sum_{i=1}^{N_z(\delta)} \hat{\sigma}_{\text{gg}}^i(t;\delta), \tag{4}$$

$$\hat{\sigma}_{\text{ee}}(z,t;\delta) = \frac{1}{N_z(\delta)} \sum_{i=1}^{N_z(\delta)} \hat{\sigma}_{\text{ee}}^i(t;\delta), \tag{5}$$

and

$$\hat{\sigma}_{\text{eg}}(z,t;\delta) = \frac{1}{N_z(\delta)} \sum_{i=1}^{N_z(\delta)} \hat{\sigma}_{\text{eg}}^i(t;\delta)e^{-i\omega_p(t-z_i/c)}. \tag{6}$$

Here, $\omega_0 = \omega_p$ is the central frequency of the inhomogeneously broadened atomic ensemble and δ denotes the detuning of different resonances in the ensemble with respect to ω_p, where we assume that the number of atoms in mode δ at z, $N_z(\delta)$, is much larger than 1. For our scheme, $N(\delta)$ is the number of atoms in the inhomogeneously broad ensemble with a periodic absorption feature (in the frequency domain) with periodicity $\mathit{\Delta}_m$ that is required for AFC storage.

Using the Heisenberg equation, one can find the following dynamical equations for the above collective operators,

$$\dot{\hat{\sigma}}_{\mathrm{gg}}(z,t;\delta) = i\Omega^*(z,t)\hat{\sigma}_{\mathrm{ge}}(z,t;\delta) - i\Omega(z,t)\hat{\sigma}_{\mathrm{eg}}(z,t;\delta), \tag{7}$$

$$\dot{\hat{\sigma}}_{\mathrm{ee}}(z,t;\delta) = i\Omega(z,t)\hat{\sigma}_{\mathrm{eg}}(z,t;\delta) - i\Omega^*(z,t)\hat{\sigma}_{\mathrm{ge}}(z,t;\delta), \tag{8}$$

and

$$\dot{\hat{\sigma}}_{\mathrm{eg}}(z,t;\delta) = i(\omega_0+\delta-\omega_p)\hat{\sigma}_{\mathrm{eg}}(z,t;\delta) + i\Omega^*(z,t)\hat{\sigma}_{\mathrm{ee}}(z,t;\delta) - i\Omega^*(z,t)\hat{\sigma}_{\mathrm{gg}}(z,t;\delta), \tag{9}$$

The propagation of the probe field can be derived starting from Maxwell's equations,

$$\left(\partial_z + \frac{n}{c}\partial_t\right)\mathcal{E}_p(z,t) = \frac{i\mu_0\omega_p^2}{2k_p}\langle\hat{\mathcal{P}}_{\mathrm{tot}}\rangle, \tag{10}$$

where $\mathcal{E}_p(z,t)$ is the slowly varying envelope of the probe field, $k_p = \frac{n\omega_p}{c}$, and $\langle\hat{\mathcal{P}}_{\mathrm{tot}}\rangle$ denotes the expectation value of

$$\hat{\mathcal{P}}_{\mathrm{tot}} = \sum_\delta \langle g|\hat{\mathbf{d}}\cdot\boldsymbol{\epsilon}_p|e\rangle\frac{N(\delta)}{V}\hat{\sigma}_{\mathrm{ge}}(z,t;\delta). \tag{11}$$

Supplementary Equations 7–11 allow us to describe the dynamics of the atoms due to the probe field for times $t < T_1$ and $t > T_2$, where T_1 and T_2 determine the expected time window for the propagating time-bin signal field. Between the probe storage and retrieval ($T_1 < t < T_2$) the evolution is perturbed by the signal field; see below. Storage of a probe field carrying an average photon number N_p that is smaller than the total number of atoms N_g is expected to result in a coherent state distribution of atomic excitations. In addition, the bosonic characteristics of the collective atomic polarization ($\hat{\sigma}_{\mathrm{ge}}(z,t;\delta)$) can be used to evaluate the above expectation value of the total atomic polarization operator, $\hat{\mathcal{P}}_{\mathrm{tot}}$.

In the proof-of-principle experiment reported in the main text, the same atomic transition is used for signal and probe fields. To minimize loss and noise for the signal, it is desirable not to have significant population in the excited state e when the signal propagates through the medium. This can be achieved either by using transitions from the same ground state to two different excited states (a V configuration), or by transferring the excited state population to another level (e.g. another ground state level or a metastable state, i.e. a Λ-type configuration) after the probe has been absorbed. The latter approach also provides a larger time window for signal propagation. By transferring the excited state population to an additional ground state, the signal sees an $\mathrm{e} \leftrightarrow \mathrm{g}$ transition without any population in e, where the number of atoms in g is reduced with respect to the total initial number by the number of probe photons that were absorbed. For optimum phase sensitivity the number of absorbed photons N_p should be of order $N/2$, where N is the total number of atoms in the AFC. In this case the number of atoms remaining in the ground state N_g is equal to N_p, $N_g = N_p = N/2$.

Derivation of effective Hamiltonian for cross-phase modulation. In this section, we provide the quantum-mechanical Hamiltonian for the interaction between the signal field and the atoms. For large detunings, where Δ is larger than the signal bandwidth, we derive an effective interaction Hamiltonian that will be used to find the probe phase shift with respect to the number of photons in the signal field.

The total Hamiltonian that governs the dynamics due to the presence of the signal field is given by

$$\hat{H}_{\text{tot}} = \hat{H}_0 + \hat{H}_{\text{int}} = \sum_{j=1}^{N} \hbar\omega_{\text{ge}}^{j}\sigma_{\text{ee}}^{j} + \hat{h}_{\text{int}}^{j}, \tag{12}$$

where

$$\hat{h}_{\text{int}}^{j} = -\hbar g\sqrt{\frac{L}{2\pi c}} \int d\omega \hat{a}_{\omega} e^{i\omega z_j/c}\hat{\sigma}_{\text{eg}}^{j} + \text{H.c.}. \tag{13}$$

As described in the earlier section on probe storage, the transition frequency of the jth atom is ω_{eg}^{j}, and the atomic coherence and population operators are denoted by $\hat{\sigma}_{\nu\nu'}^{j} = |\nu\rangle^{j}\langle\nu'|$, where $\nu, \nu' = \{g, e\}$. $\hat{a}_{\omega}$ is the anihilation operator for signal field modes and the single photon coupling is given by $g = \mu_{\text{eg}}\sqrt{\frac{\omega_s}{2\hbar\epsilon V}}$, where ω_s is the central frequency of the signal, and the transition dipole moment is $\mu_{\text{eg}} = \langle e|\mathbf{d}\cdot\boldsymbol{\epsilon}_s|g\rangle$. Throughout this analysis, we assume that $\omega_p = \omega_0$, where ω_0 is the central frequency of the AFC.

We use the collective atomic operators that are defined in Supplementary Equations 4 – 6 to re-write the interaction Hamitonian in Supplementary Equation 13. This results in

$$\hat{H}_{\text{int}} = -\hbar g \sum_{j=1}^{N} \sqrt{\frac{L}{2\pi c}} e^{i\omega_p(t-z_j/c)} \int d\omega \hat{a}_{\omega} e^{i\omega z_j/c}\hat{\sigma}_{\text{eg}}^{j} e^{-i\omega_p(t-z_j/c)} + H.c., \tag{14}$$

which leads to

$$\hat{H}_{\text{int}} = -\hbar g \int dz n_z(\delta) e^{i\Delta(t-z/c)} \hat{\mathcal{E}}_s(z,t)\hat{\sigma}_{\text{eg}}(z,t;\delta) + H.c., \tag{15}$$

where $\hat{\mathcal{E}}_s(z,t) = \sqrt{\frac{L}{2\pi c}} e^{i\omega_s(t-z/c)} \int d\omega \hat{a}_{\omega} e^{i\omega z/c}$, $n_z(\delta)dz = N_z(\delta)$, $\int dz n_z(\delta) = N(\delta)$ and $\Delta = \omega_p - \omega_s$ is the detuning between the signal and probe fields.

For detunings much larger than the bandwidth of the signal field ($\Delta \gg 1/\tau_s$), we expect the dynamics of the collective atomic polarization ($\hat{\sigma}_{\text{eg}}(z,t;\delta)$) to be dominated by the fast rotating terms of $e^{\pm i\Delta t}$. In order to capture this effect, let us consider the dynamics of $\hat{\sigma}_{\text{eg}}(z,t;\delta)$ due to $\hat{H}_{\text{int}}$. Starting from $\dot{\hat{\sigma}}_{\text{eg}}(z,t;\delta) = \frac{i}{\hbar}\left[\hat{H}_{\text{int}}, \hat{\sigma}_{\text{eg}}(z,t;\delta)\right]$, we find

$$\dot{\hat{\sigma}}_{\text{eg}}(z,t;\delta) = -ige^{-i\Delta(t-z/c)}\hat{\mathcal{E}}_s^{\dagger}(z,t)\left(\hat{\sigma}_{\text{gg}}(z,t;\delta) - \hat{\sigma}_{\text{ee}}(z,t;\delta)\right), \tag{16}$$

which leads to

$$\hat{\sigma}_{\text{eg}}(z,t;\delta) = -ig\int_0^t dt' e^{-i\Delta(t'-z/c)}\hat{\mathcal{E}}_s^{\dagger}(z,t')\left(\hat{\sigma}_{\text{gg}}(z,t';\delta) - \hat{\sigma}_{\text{ee}}(z,t';\delta)\right). \tag{17}$$

For any state and for large detunings ($\Delta \gg 1/\tau_s$), this integral can be approximately evaluated by integrating the fast oscillating part and multiplying it by the final value of the slowly varying component. This approximation allows us to find the collective atomic polarization as

$$\hat{\sigma}_{\rm eg}(z,t;\delta) = \frac{g}{\Delta} e^{-i\Delta(t-z/c)} \hat{\mathcal{E}}_s^{\dagger}(z,t) \left(\hat{\sigma}_{\rm gg}(z,t;\delta) - \hat{\sigma}_{\rm ee}(z,t;\delta)\right). \tag{18}$$

Using the above equation, in an iteration, we replace $\hat{\sigma}_{\rm eg}(z,t;\delta)$ in Supplementary Equation 15 to find an effective interaction Hamiltonian as follows,

$$\hat{H}_{\rm int}^{\rm eff} = -\frac{\hbar g^2}{\Delta} \int dz n_z(\delta) \left(\hat{\mathcal{E}}_s(z,t)\hat{\mathcal{E}}_s^{\dagger}(z,t) + \hat{\mathcal{E}}_s^{\dagger}(z,t)\hat{\mathcal{E}}_s(z,t)\right) \left(\hat{\sigma}_{\rm gg}(z,t;\delta) - \hat{\sigma}_{\rm ee}(z,t;\delta)\right). \tag{19}$$

Using the effective interaction Hamiltonian we derive the dynamical equation for the collective atomic polarization.

$$\dot{\hat{\sigma}}_{\rm eg}(z,t;\delta) = \frac{i}{\hbar}\left[\hat{H}_0 + \hat{H}_{\rm int}^{\rm eff}, \hat{\sigma}_{\rm eg}(z,t;\delta)\right] + \frac{\partial \hat{\sigma}_{\rm eg}(z,t;\delta)}{\partial t}.$$

This leads to

$$\dot{\hat{\sigma}}_{\rm eg}(z,t;\delta) = i\delta\hat{\sigma}_{\rm eg}(z,t;\delta) + \frac{2ig^2}{\Delta}\left(\hat{\mathcal{E}}_s(z,t)\hat{\mathcal{E}}_s^{\dagger}(z,t) + H.c.\right)\hat{\sigma}_{\rm eg}(z,t;\delta), \tag{20}$$

and consequently

$$\hat{\sigma}_{\rm eg}(z,t=T_2;\delta) = e^{i\delta t} e^{i\hat{\Phi}} \hat{\sigma}_{\rm eg}(z,t=T_1;\delta), \tag{21}$$

where

$$\hat{\Phi} = \int_{T_1}^{T_2} dt' \tfrac{2g^2}{\Delta}\left(\hat{\mathcal{E}}_s(z,t')\hat{\mathcal{E}}_s^{\dagger}(z,t') + \hat{\mathcal{E}}_s^{\dagger}(z,t')\hat{\mathcal{E}}_s(z,t')\right). \tag{22}$$

Note that the above equations describe the effects during the signal field propagation. The storage and retrieval of the probe field can be treated separately. As it has been discussed in Supplementary Reference 1, a frequency offset such as the one shown in Supplementary Equation 21 leads to a phase factor for the re-emitted probe field. Therefore, phase modulations due to the presence of the signal will appear in the first echo of the probe field. In addition, the total phase only depends on the total energy in the signal field and does not reveal any information about the temporal distribution of the signal field.

Phase shift per signal photon. Given Supplementary Equations 21 and 22, one can find the total phase shift for a single-photon signal propagating in the waveguide and interacting off-resonantly with the atomic polarization. The electric dipole interaction Hamiltonian can be used to relate the spontaneous emission rate of a two-level system to its transition dipole moment. For two-level atoms in a solid with dipoles oriented along a specific direction, for emission polarized along the dipoles, this results in $\gamma = \frac{\mu_{\rm eg}^2 \omega_0^3}{\pi\epsilon\hbar c^3}$ (One can consider the local field effects in the relation for

spontaneous emission rate where for an index of refraction near 2, this is expected to introduce relatively small corrections to the current treatment [2]).

Assuming that $\lambda_s \approx \lambda_0$ we can find the phase shift due to a single photon as

$$\phi = \frac{2g^2}{\mathit{\Delta}}\tau_s = \frac{1}{4\pi}\frac{\lambda_0^2}{n^2 A}\frac{\gamma}{\mathit{\Delta}}, \tag{23}$$

where $\tau_s = L/c$ is the duration of the signal in vacuum, A is the transverse mode area of the interaction, and λ_0 is the wavelength associated with the corresponding atomic transition in vacuum. If the population in the excited state is transferred to another ground state in order to minimize loss and noise (as discussed at the end of the earlier section on probe storage), then this expression for the phase shift has to be divided by a factor of 2 because this second ground state is unaffected by the AC Stark shift due to the signal.

Signal loss. In this section, we analyze the signal loss due to its off-resonant interaction with the atoms in the AFC. In order to find a simplified description for off-resonant absorption loss, we assume that the signal detuning is larger than the inhomogenous bandwidth of the ensemble. This also guarantees that $\mathit{\Delta}$ is much larger than the spontaneous emission rate, γ.

To analyze the signal loss, we treat the signal propagation using the Maxwell equation as follows

$$\left(\partial_z + \frac{n}{c}\partial_t\right)\hat{\mathcal{E}}_s(z,t) = \frac{\mu_0\omega_0^2\mu_{\text{eg}}}{2k_0}\sum_\delta \frac{N(\delta)}{V}\hat{\sigma}_{\text{eg}}(z,t;\delta), \tag{24}$$

where $k_0 = \frac{n\omega_0}{c}$. Given that the equations governing the single-excitation wave functions are the same as the Maxwell-Bloch equations, for evaluating the signal loss, we take Fourier transform of the Maxwell-Bloch equations for single-excitation wavefunctions. As a result the output signal wave function in the frequency domain is given by

$$\tilde{\mathcal{E}}_s(z,\omega)|_{z=L} = e^{ik_s\chi(\omega)L}\tilde{\mathcal{E}}_s(z=0,\omega), \tag{25}$$

where

$$\chi(\omega) = \frac{1}{k_s}\left(-\frac{n\omega}{c} + \frac{\mu_0\omega_0^2\mu_{\text{eg}}}{2k_0}\sum_\delta \frac{N(\delta)}{V}\frac{i\mu_{\text{eg}}/2\hbar}{i(\omega-(\mathit{\Delta}+\delta))-\gamma}\right), \tag{26}$$

and $k_s = \frac{n\omega_s}{c}$. The imaginary part of $\chi(\omega)$ determines the loss. We can simplify the above expression by assuming $\mathit{\Delta} \gg \mathit{\Gamma}$, where $-\mathit{\Gamma}/2 < \delta < \mathit{\Gamma}/2$. In addition, the loss is expected to be uniform over the signal field spectrum when its bandwidth is smaller than the detuning ($\mathit{\Delta} > 1/\tau_s$). This results in a rather simple expression for the imaginary part of the response function, $\chi(\omega)$. For $\mathit{\Delta} \gg \gamma$, it is given by

$$\text{Imag}(\chi(0)) = \frac{1}{k_s}\frac{1}{16\pi}\frac{N_g\lambda_0^2\gamma^2}{n^2 V\mathit{\Delta}^2}. \tag{27}$$

Therefore, the intensity loss for the signal field can be derived from

$$|\mathcal{E}(L,\omega)|^2 = e^{-\zeta L}|\mathcal{E}(0,\omega)|^2, \tag{28}$$

where

$$\zeta L = \frac{1}{8\pi}\frac{N_g\lambda_0^2}{n^2 A}\frac{\gamma^2}{\varDelta^2}. \tag{29}$$

Requirements for single-photon sensitivity and low loss; multipass arrangement. Single photon sensitivity requires $\sqrt{\eta N_p}\phi > 1$, with ϕ given by Supplementary Equation 23 with an additional factor $1/2$ assuming that the excited state population is transferred to another ground state to minimize loss and noise. Here η is the retrieval efficiency of the probe field in the AFC [1],

$$\eta = (1 - e^{-d/F})^2 e^{-\frac{\pi^2}{2\ln 2 F^2}}, \tag{30}$$

where d and $F = \varDelta_m/\gamma$ are optical depth and finesse of the AFC, respectively. Assuming $N_p = N_g$, as discussed in the first subsection of Supplementary Note 1, this puts a lower bound on the number of atoms in g,

$$N_g > \frac{1}{\eta\phi^2} = \frac{1}{\eta}\left(\frac{8\pi n^2 A\varDelta}{\lambda_0^2\gamma}\right)^2, \tag{31}$$

and hence, using Supplementary Equation 29, a lower bound on the loss experienced by the signal:

$$\zeta L = \frac{N_g\lambda_0^2\gamma^2}{8\pi n^2 A\varDelta^2} > \frac{8\pi n^2 A}{\eta\lambda_0^2}. \tag{32}$$

Even for very small cross sections of order λ_0^2/n^2, this loss is $8\pi/\eta \gg 1$, which is too high for a nondestructive measurement. A similar limitation has also been pointed out for generating giant Kerr nonlinearity based on electromagnetically-induced transparency [3], and in the context of nondestructive detection of atoms in Bose-Einstein condensates [4].

This problem can be overcome by using a multipass arrangement, where m is the number of passes the signal makes through the medium. In this case the phase shift ϕ in Supplementary Equation 23 and the relation for the loss ζL in Supplementary Equation 29 are both multiplied by m. However, the lower bound on N_g of Supplementary Equation 31 that originates from the single photon sensitivity requirement scales as $1/\phi_{\text{tot}}^2$, where $\phi_{\text{tot}} = m\phi$. Therefore, the lower bound on N_g of Supplementary Equation 31 is multiplied by $\frac{1}{m^2}$, which finally leads to a modified bound on the total loss,

$$m\zeta L > \frac{8\pi n^2 A}{m\eta\lambda_0^2}, \tag{33}$$

which can be much less than one for sufficiently many passes. Requiring small total signal loss $m\zeta L \lesssim 0.1$, Supplementary Equation 33 gives a condition on m,

$$m > 80\pi/\eta, \tag{34}$$

where we have assumed a small waveguide, $A = \lambda_0^2/n^2$.

Implementing $m \gg 1$ in practice requires low-loss switches. However, an analogous effect can also be achieved by using a cavity. The main difference is that a cavity enhances the signal

field rather than the interaction time, which reduces the requirements on the storage time for the probe compared to a multi-pass scenario. Here we focus on the multi-pass case for simplicity.

For a small waveguide as above, and for an AFC where each tooth corresponds to one radiatively broadened line, the optical depth per atom for each absorption line is $\lambda^2/n^2 A \approx 1$. This leads to number of atoms in each AFC tooth to be equal to the optical depth and therefore having the total number of atoms in the inhomogeneously broadened ensemble N as $N = n_t d$, where n_t is the number of teeth in the comb. This leads to another condition that follows from Supplementary Equation 31, which in the multi-pass case, it can be rewritten as

$$d > \frac{128\pi^2 \varDelta^2}{n_t \gamma^2 m^2 \eta}, \tag{35}$$

where in deriving Supplementary Equation 35 we have again assumed $N_g = N/2$. Supplementary Equation 35 yields a condition on the number of passes,

$$m > \frac{8\sqrt{2}\pi\varDelta}{\sqrt{n_t \eta d}\gamma}. \tag{36}$$

We now rewrite the detuning $\varDelta = f n_t F \gamma$, where $F = \varDelta_m/\gamma$ is the finesse of the AFC and f is a factor greater than one that assures that the signal is sufficiently far detuned from the AFC (whose total width is $n_t F \gamma$). This yields

$$m > 8\sqrt{2}\pi f F \sqrt{\frac{n_t}{d\eta}}. \tag{37}$$

Supplementary Equation 36 also yields a condition on the number of passes as a function of the desired signal bandwidth B. For B expressed in Hz one has $B = \varDelta/(2\pi f)$, where the factor f again ensures that the signal is off-resonant. This gives

$$m > \frac{16\sqrt{2}\pi^2 f B}{\sqrt{n_t \eta d}\gamma} \tag{38}$$

For our material system (Thulium ions in Lithium Niobate, which have γ of approximately 9 kHz) all the above conditions are satisfied, for example, by setting $f = 3$, $d = 30$ as achieved in Ref. 5, $B = 500$ kHz, $F = 3.2$, $n_t = 110$ and $m = 930$. The latter is probably impossible for multiple passes using switches, but corresponds to only a moderate-finesse cavity [6]. For smaller bandwidth and higher optical depth smaller values of m are sufficient, but note that Supplementary Equation 34 implies $m > 80\pi$ under all circumstances. The bandwidth can be increased by working with a system that has a larger γ, such as Ce ions in appropriate crystals [7,8] or colour centres in diamond.

Supplementary References

1. Afzelius, M., Simon, C., de Riedmatten, H. & Gisin, N. Multimode quantum memory based on atomic frequency combs. *Phys. Rev. A* **79**, 052329 (2009).

2. Sun, Y., Thiel, C.W., & Cone, R.L., Optical decoherence and energy level structure of 0.1%Tm^{3+}:$LiNbO_3$. *Phys. Rev. B* **85**, 165106 (2012).

3. Gea-Banacloche, J. Impossibility of large phase shifts via the giant Kerr effect with single-photon wave packets. *Phys. Rev. A* **81**, 043823 (2010).

4. Hope, J.J. & Close, J.D. General limit to nondestructive optical detection of atoms. *Phys. Rev. A* **71**, 043822 (2005).

5. Hedges, M.P., Longdell, J.J., Li, Y. & Sellars, M.J. Efficient quantum memory for light. *Nature* **465**, 1052–1056 (2010).

6. Zhong, T., Kindem, J.M., Miyazono, E. & Faraon, A. Nanophotonic coherent light-matter interfaces based on rare-earth-doped crystals. *Nat. Commun.* **6**, 8206 (2015).

7. Kolesov, R. *et al.* Mapping spin coherence of a single rare-earth ion in a crystal onto a single photon oolarization state. *Phys. Rev. Lett.***111**, 120502 (2013).

8. Karlsson, J. *et al.* High resolution transient and permanent spectral hole burning in Ce^{3+}:Y_2SiO_5 at liquid helium temperatures. *Phys. Rev. B* **93**, 224304 (2016).
